# Phytoplankton in Deep Lakes of the Dinaric Karst: Functional Biodiversity and Main Ecological Features

**DOI:** 10.3390/plants13162252

**Published:** 2024-08-14

**Authors:** Nikola Hanžek, Mario Šiljeg, Tanja Šikić, Igor Stanković

**Affiliations:** Josip Juraj Strossmayer Water Institute, Ulica grada Vukovara 220, 10000 Zagreb, Croatia; nikola.hanzek@institutjjs.hr (N.H.); mario.siljeg@institutjjs.hr (M.Š.); tanja.sikic@institutjjs.hr (T.Š.)

**Keywords:** Reynolds functional groups, chlorophyll *a*, salinity, nutrients, trophic status, water quality monitoring

## Abstract

Phytoplankton is a polyphyletic group of organisms that responds rapidly to environmental conditions and provides a reliable response to changes, making it a good ecological indicator for water quality monitoring. However, a gradient is almost essential for a reliable relationship between pressure and impact. In a low-gradient environment, ingenuity is required to outsmart the limitations of the commonly used linear relationship. Here, we examine changes in biomass and functional biodiversity by analysing larger data sets (2013–2022) in six ecologically diverse, natural, deep Croatian karst lakes with low nutrient gradients using nonlinear correlation coefficients and multivariate analyses in 209 samples. We found that phytoplankton biomass was most strongly influenced by nutrients, salinity and alkalinity, while light availability and total nitrogen strongly influenced phytoplankton functional biodiversity. An additional analysis of the TN:TP ratio revealed that the oligotrophic Lake Vransko is nitrogen-limited, and lakes Kozjak and Prošće are phosphorus-limited. This further clarified the relationship of phytoplankton to nutrients despite the low gradient. The complex analysis in this study provides a new perspective for predicting changes in the structure and succession of phytoplankton in deep karst lakes for successful management under apparent anthropogenic pressure and climate change.

## 1. Introduction

Freshwater lakes, which are rich in biodiversity, are vulnerable to human impacts, with climate change and nutrient fluctuations being among the main drivers of change in these ecosystems. One of the consequences of these factors is eutrophication [1]. As lakes are complex ecosystems, they respond rapidly to environmental changes related to their physico-chemical and biological properties. The process of mixing and stratification also plays an important role in the ecological properties of lake water, in particular, water temperature, dissolved oxygen consumption and the nutrient content and distribution of phytoplankton and its diversity in the layers of the lake [2,3,4].

Nitrogen (N) and phosphorus (P) are essential macronutrients that are crucial for the biochemical processes of phytoplankton and, as limiting factors in the N:P ratio, determine the growth dynamics of phytoplankton in aquatic ecosystems, including lakes [5]. Understanding the N:P ratio is crucial for explaining and predicting phytoplankton dynamics in different aquatic habitats and is of great importance for the effective management and maintenance of ecological balance in lakes and other aquatic ecosystems [6,7,8]. Since oligotrophic lakes have low nutrient concentrations, they are sensitive to even small changes in the N or P supply. As a result of the low N and P concentration, the naturally low N:P ratio in oligotrophic lakes can increase considerably with high N inputs. The phytoplankton in these lakes can change from a primarily nitrogen-limited to a primarily phosphorus-limited form [9,10]. An increased N:P ratio can lead to reduced biodiversity in the lake’s food web, lower drinking water quality and algal blooms [11]. In addition to the main factors N and P, silicates are also of great importance for the growth of phytoplankton in freshwater ecosystems, especially for diatoms [12].

Not only nutrients are important for phytoplankton communities, but other factors also determine their dynamics, including water temperature, salinity, light availability, alkalinity, pH, suspended matter, hydrological characteristics and human activities [13,14,15,16,17]. Recognising that phytoplankton is an extremely diverse group of organisms, which makes it difficult to understand ecological processes and the influence of ecological indicators on phytoplankton, Reynolds developed the concept of functional groups based on the common ecology and environmental preferences of phytoplankton species [18,19,20].

Due to the great heterogeneity and variability in geological, morphological, hydrogeological, hydrological, hydraulic, ecological and other parameters, an interdisciplinary approach is required for the study of karst systems. A key feature of the karst phenomenon is the activity of groundwater and surface water, which influences biological processes both on the surface and underground [21]. Phytoplankton, one of the most important biological elements in freshwater ecosystems, plays an important role in the ecology of karst lakes and is thus an essential component of these complex interactions in such environments.

Stratified karst lakes are unique due to their geological, physical and chemical characteristics associated with karst landscapes. Such lakes in this study are mostly in an oligo- and mesotrophic state and are characterised by the presence of the phytoplankton groups Ochrophyta, Miozoa and Bacillariophyta, which are the most diverse and abundant [22,23]. Since they are mostly in a pristine state, they represent ecosystems that can be used to study changes in the phytoplankton community under the influence of humans and the resulting climate changes [24], as phytoplankton responds quickly and reliably to environmental changes and is a good ecological indicator of the state of nutrients and eutrophication [14,25].

## 2. Results

### 2.1. Environmental Characteristics of Lakes

**Figure 1:**
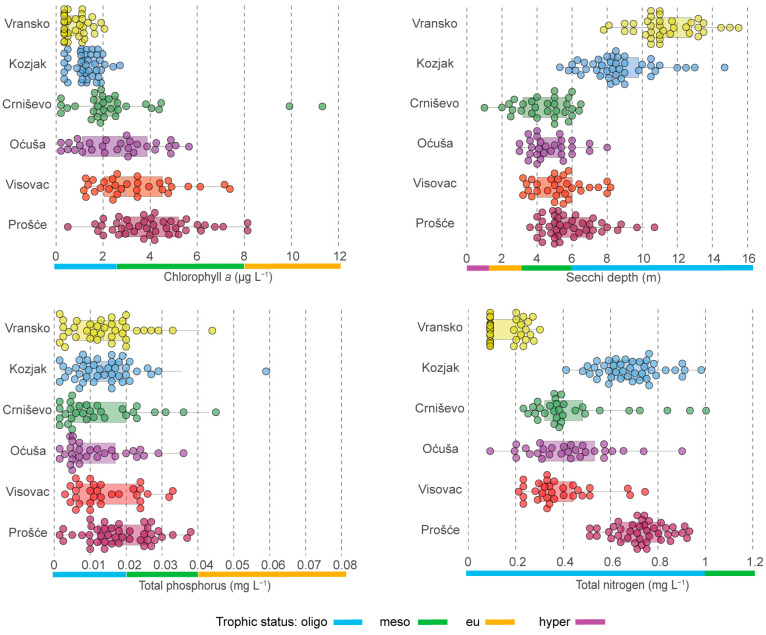
Classification of trophic state according to chlorophyll *a*, Secchi depth, total phosphorus and total nitrogen, shown as boxplots, with the values for each trophic state indicated by the colours below the x-axis. The centre line shows the median value, while outliers are shown as dots.

The minimum, maximum and mean values for the physical and chemical properties of the water in all six lakes are shown in Table 1. The Secchi depth in the deep karst lakes ranged from 1.0 to 15.5 m, with the lowest values measured in Lake Crniševo and the highest in Lake Vransko, indicating an oligotrophic state for lakes Kozjak and Vransko, an oligo- to eutrophic state for Lake Crniševo and an oligo-mesotrophic state for all other lakes (Figure 1). The continental lakes Prošće and Kozjak were the coldest with a mean temperature of 12.8 and 13.3 °C, respectively, while the highest values were measured in the Mediterranean lakes with a mean temperature between 17.7 and 22.6 °C. The analysed lakes had a slightly alkaline character, with mean pH values of 8.1 to 8.3. Lake Prošće was the richest in dissolved oxygen, and Lake Visovac had the highest saturation, while Lake Oćuša was characterised by the lowest concentration of dissolved oxygen and the lowest saturation. The highest salinity and consequently the highest conductivity values were measured in the slightly brackish Lake Crniševo. In terms of nutrients, Lake Vransko had the lowest TN values (0.100-0.310 mg L^−1^), while the highest TN values were measured in lakes Kozjak and Prošće with mean values of 0.685 and 0.740 mg L^−1^, respectively (Figure 1). The TN values indicate an oligotrophic state of all lakes. The mean values of TP showed the lowest concentrations in lakes Oćuša and Crniševo (0.007 and 0.009 mg P L^−1^), while the highest mean value was measured in Lake Prošće (0.018 mg P L^−1^), indicating an oligotrophic state for all analysed lakes (Figure 1). The organic load, measured as the BOD and COD, was higher in the lakes Visovac, Crniševo and Oćuša (0.1–2.7 mg O_2_ L^−1^) than in the lakes Kozjak, Prošće and Vransko (0.3–1.8 mg O_2_ L^−1^). The TOC values measured in all lakes were between 0.65 and 3.70 mg C L^−1^ with the lowest values in the Kozjak, Prošće and Visovac barrage lakes and the highest in the Crniševo and Vransko lakes. The SiO_2_ concentration in all lakes was between 0.13 and 4.96 mg L^−1^ with the lowest average values in lakes Vransko and Crniševo (0.35 and 0.70 mg L^−1^) and the highest in all other lakes (1.52–1.95 mg L^−1^).

**Figure 2:**
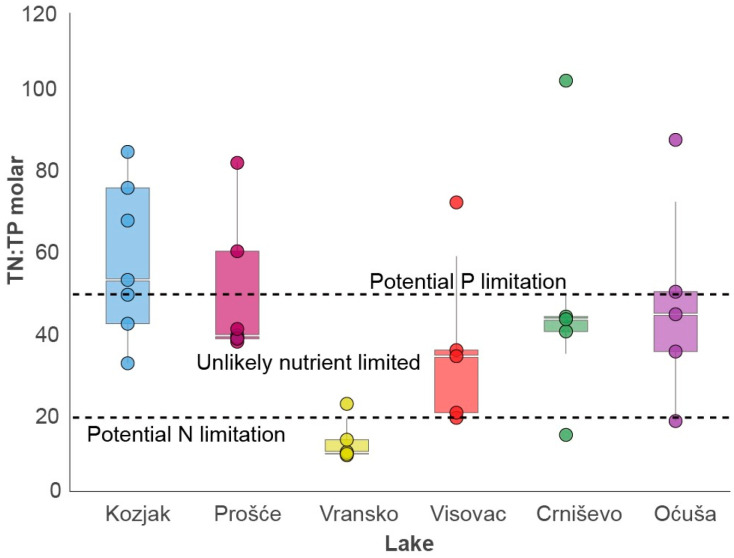
Boxplots of the average molar ratios TN:TP per lake. The lines show the TN:TP molar ratios at which N and P limitation can occur: <20 molar ratio N limitation; >50 molar ratio P limitation [26].

The molar ratio TN:TP varied between the lakes (Figure 2). The lowest mean TN:TP ratio was calculated for Lake Vransko (14.8), which is thus categorised as a potentially N-limited lake. The highest ratios were determined in lakes Kozjak and Prošće (62.5 and 48.6), which categorises these lakes as potentially P-limited. The mean ratio values for lakes Visovac, Crniševo and Oćuša lie between the N- and P-limitation lines, indicating that these lakes are probably not nutrient-limited.

**Figure 3:**
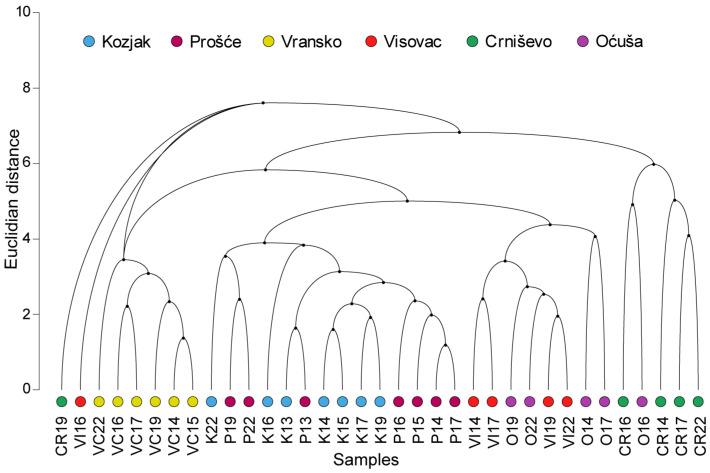
Dendrogram of the cluster analysis based on the Euclidean distance of the physical and chemical properties of the water in lakes. The lakes are coded with different coloured symbols, while the two attached numbers represent the years of the study from 2013 to 2022. The location codes of the lakes are shown in Figure 1.

Cluster analysis of the Euclidean distance of water physical and chemical properties (including Secchi depth, alkalinity, conductivity, pH, salinity, temperature, BOD, COD, dissolved oxygen, saturation, nitrates, nitrites, TN, soluble reactive phosphorus, TP, TOC and SiO_2_) was performed based on the average data for each lake. The analysis resulted in a clear grouping of lakes, although there were some exceptions (Figure 3). Lake Vransko formed a separate group. Lakes Kozjak and Prošće were grouped together. However, lakes Oćuša and Crniševo, both part of the Baćina Lakes complex, were not grouped together, with Lake Crniševo forming its own group for the majority of years. Lakes Oćuša and Visovac consistently formed a common group, also with minor exceptions.

### 2.2. Phytoplankton Biomass and Its Relationship to Environmental Parameters

Chl-a values showed that lakes Vransko and Kozjak were the least productive lakes with average values of 0.5 and 1.29 µg L^−1^, respectively. The productivity of the other lakes was in the following order: Crniševo, Visovac, Oćuša and Prošće. The highest mean Chl-a was measured in Lake Prošće at 4.01 µg L^−1^. Lakes Vransko, Kozjak and Crniševo had an oligotrophic status, while Oćuša, Visovac and Prošće had a mesotrophic status according to the mean Chl-a values (Figure 1).

Spearman correlation analysis of all 15 environmental parameters showed a similar relationship between the environmental variables and phytoplankton biomass, represented by Chl-a and total biomass (Table 2). The alkalinity, conductivity, salinity, TN, SiO_2_ and presence of organic matter, represented by BOD, showed a significant positive relationship with both Chl-a and total biomass, while the Secchi depth and pH showed a significant negative relationship for both factors. TP showed a significant positive correlation, while TOC showed a significant negative correlation with phytoplankton biomass. COD and TN:TP showed a significant positive correlation with Chl-a, while dissolved oxygen and saturation showed a significant negative correlation with Chl-a.

### 2.3. The Functional Composition of Phytoplankton

A total of 341 phytoplankton taxa were identified in 209 samples from six deep karst lakes. These taxa were categorised into ten main groups (Phyla), Chlorophyta (115), Bacillariophyta (66), Cyanobacteria (60), Ochrophyta (46), Charophyta (16), Miozoa (13), Cryptophyta (9), Euglenozoa (11), Haptophyta (3) and Choanozoa (2), and classified into 25 coda of Reynolds’ FGs. The complete taxa list for each lake is included in the Supplementary Table S1.

The one-way SIMPER analysis based on the Bray–Curtis similarity of phytoplankton FGs showed that 12 FGs contributed to more than 5% similarity depending on the lake and were thus classified as descriptive FG coda (Table 3). The most common descriptive coda found in all six lakes were **L0**, **X2** and **F**. Representatives of these FGs were codominant in the phytoplankton communities in all lakes, with the exception of the dominance of **L0** in lakes Crniševo and Vransko. The most common representatives of centric diatoms, grouped in codon **A**, were descriptive in all lakes except Lake Visovac, with the highest similarity in lakes Kozjak, Vransko and Oćuša. Centric diatoms belonging to coda B and D together contributed the most to the similarity of samples in lakes Kozjak and Prošće. In addition, codon **B** was also descriptive in lakes Visovac and Vransko, while it dominated in lakes Prošće and Visovac. Coda **E** and **Y** were codominant in lakes Kozjak, Prošće and Oćuša. The FG coda that were specific to a single lake were **T** (Vransko), **X3** (Visovac), **X1** (Crniševo) and **J** (Oćuša). 

**Figure 4:**
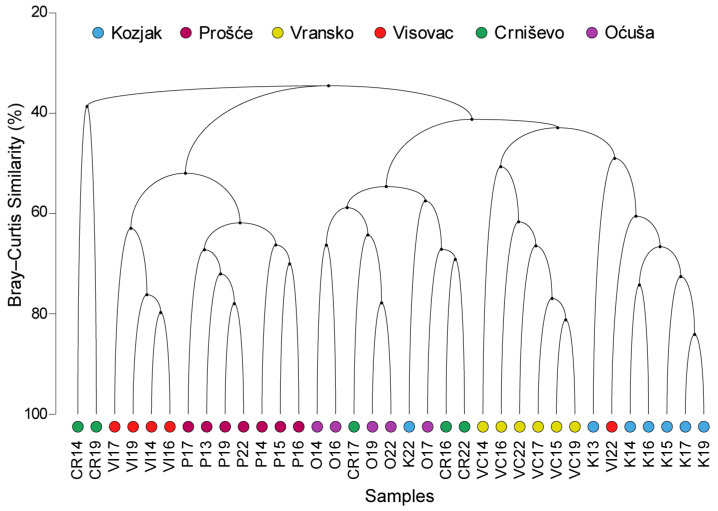
Dendrogram of the cluster analysis based on the Bray–Curtis similarity index of phytoplankton FG composition. The lakes are coded with different coloured symbols, while the two attached numbers represent the years of the study from 2013 to 2022. The location codes of the lakes are shown in Figure 1.

The cluster analysis of the Bray–Curtis similarity of the composition of the functional groups based on the average biomass data of the lakes revealed a clear grouping according to the indicated trophic status of the lakes, with minor exceptions (Figure 4). The least productive lakes Vransko and Kozjak were grouped in a cluster with a similarity of over 40%. Both lakes were in a larger cluster with the slightly more productive lakes Oćuša and Crniševo, while the most productive lakes Prošće and Visovac were grouped separately with minor exceptions.

### 2.4. Relationship between Environmental Parameters and the Composition of Phytoplankton

**Figure 5:**
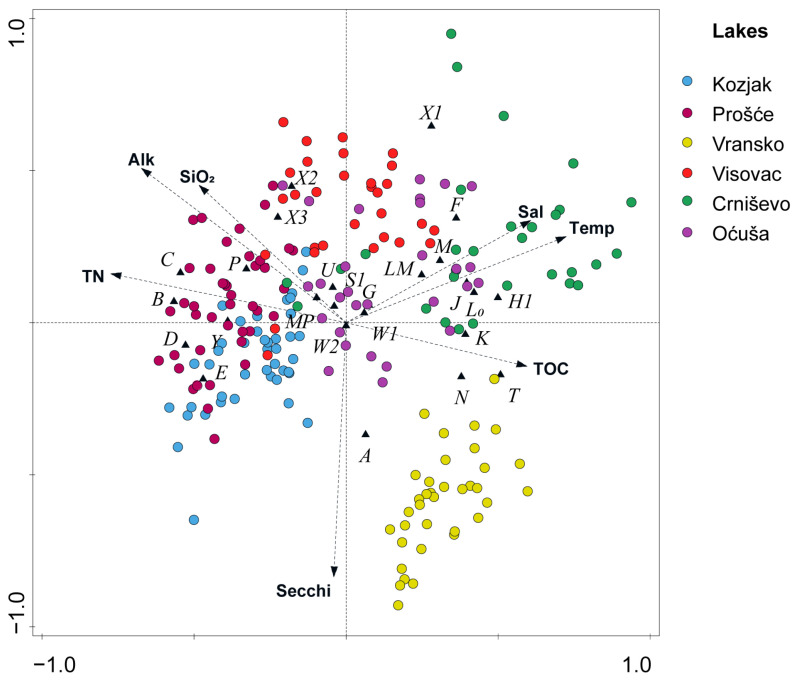
Redundancy analysis (RDA) between FGs and environmental variables for lakes throughout the study period. Codes of the variables: T—water temperature; Sal—salinity; Secchi—Secchi depth; Alk—alkalinity; TN—total nitrogen; SiO_2_—silicates; and TOC—total organic carbon.

The ordination diagram of the redundancy analysis (RDA) of the FG composition of the phytoplankton and the environmental variables is shown in Figure 5. The environmental variables with a significant influence on the phytoplankton composition for deep karst lakes were the TN, alkalinity, SiO_2_, salinity, temperature, TOC and Secchi depth. The eigenvalues of the first two axes were 0.163 and 0.058, respectively, explaining 76.1% of the relationship between FGs and environmental data (Table 4). Axis 1 had the highest correlation with TN, while axis 2 had the highest correlation with Secchi depth. Codon **A** favoured conditions with more light, especially in lakes Vransko and Kozjak, while in lakes Visovac and Oćuša, coda **X3** and **X2** favoured less light. In lakes Kozjak and Prošće, codons **B**, **C**, **D** and **P** favoured conditions with higher TN, alkalinity and SiO_2_. The highest salinity and temperature characterised Lake Crniševo, which favoured coda **H1**, **L0** and **J**. However, TOC also characterised Lake Crniševo, together with Lake Vransko, which mostly favoured coda **K**, **N** and **T**. Codon **F** favoured conditions with low light and low TN at higher temperature and salinity.

## 3. Discussion

In this study, we have found that the response of phytoplankton biomass and composition to environmental variables is different for each lake, as all lakes are unique habitats. However, it can be generalised that the environmental variables with the greatest influence on phytoplankton are nutrients, alkalinity, salinity, light availability and water temperature. These results suggest that there is a close relationship between phytoplankton and environmental variables, making phytoplankton a reliable biological indicator of the response to nutrients and eutrophication in freshwater lakes, especially on the threshold of climate change. 

### 3.1. Physical and Chemical Properties of the Analysed Lakes and Indicators of Trophic Status

The higher TN concentrations in the Kozjak and Prošće lakes compared to the other studied lakes can be explained by higher concentrations of these variables in the spring water feeding the lakes and indicate the influence of the natural environment, e.g., seepage of groundwater through humus and transport by surface water from the forested surroundings [27]. The higher water temperatures in lakes Vransko, Visovac, Oćuša and Crniševo, which are located in the Mediterranean region, are directly related to the higher air temperatures characteristic of the Mediterranean climate [28]. In contrast, lakes Kozjak and Prošće, which are located in the mountainous continental region with its colder winters and milder summers, have lower water temperatures due to the continental climate [4,29]. 

### 3.2. Relationship between Environmental Variables and Phytoplankton Biomass

Light availability, as a crucial resource for autotrophic organisms, which is measured indirectly via the Secchi depth, has a direct influence on phytoplankton biomass [30]. However, the Secchi depth is higher in less productive lakes because the algae themselves contribute to lake turbidity [3,31,32]. This pattern was also found in the lakes studied, where low productivity means higher Secchi depth, making this parameter a good indicator of eutrophication [25]. The positive correlation of the BOD with Chl-a and total phytoplankton biomass in this study also proves that phytoplankton is a good indicator of anthropogenic influences, as the BOD is always elevated when phytoplankton blooms occur in organically enriched water from domestic or other sources [33].

The analysed lakes are in a mesotrophic and oligotrophic state. Nevertheless, Chl-a showed a negative correlation with dissolved oxygen and saturation, possibly due to the occurrence of stratification [34], where oxygen production takes place in the upper layers of the lake and at the thermocline, where more light and nutrients are available, while phytoplankton decomposition consumes oxygen in the lower layers, which could link the average oxygen values of the composite sample from the euphotic zone to the negative Chl-a. As phytoplankton biomass increases, the nocturnal periods when respiration replaces photosynthesis may also reduce dissolved oxygen and saturation. This event can also be explained by aquatic respiration and the oxidative degradation of organic compounds [35].

The importance of nutrients is critical for the growth and maintenance of phytoplankton communities [36], and nutrients are influenced by many factors, including salinity, which affects their availability, which in turn influences phytoplankton growth. N and P are the most important nutrients for growth in brackish water and estuarine ecosystems, and their concentrations, which are determined by the direct interaction of nutrients and salinity, consequently have a direct positive or negative influence on Chl-a and phytoplankton biomass [3,37,38]. This is important for our study because conductivity and salinity had a positive influence on phytoplankton growth. Their values were highest in lakes Crniševo and Oćuša, where Chl-a and total biomass were also high and showed a positive correlation with Chl-a.

An increase in alkalinity has a positive effect on Chl-a and the biomass of phytoplankton. As bicarbonate is the predominant form of carbon in freshwaters with a similar pH range to the lakes studied and its concentration often exceeds that of CO_2_, it is much more accessible to photosynthetic organisms [39]. The alkalinity values were highest in the tufa-dominated Prošće and Kozjak lakes, where the dominance of Fragilaria-like diatoms, which efficiently utilise HCO_3-_ as an inorganic carbon source, was noted [40]. Regarding the direct effects of nutrients, the availability and proportion of SiO_2_ relative to dissolved inorganic nutrients are important in regulating competition between phytoplankton species [41]. In this study, the increase in phytoplankton biomass and Chl-a was accompanied by an increase in SiO_2_. This observation is consistent with the findings of Fetahi et al. [30] and Dubourg et al. [42], as SiO_2_ is particularly crucial for diatoms, one of the most numerous and abundant phytoplankton groups addressed in this work.

In the lakes studied, the increase in phytoplankton biomass together with Chl-a is accompanied by an increase in TN concentrations, while an increase in TP only contributes to phytoplankton biomass, confirming the importance of both N and P for phytoplankton growth and primary production in lakes [43,44]. P is widely considered to be the most important factor influencing phytoplankton growth [45], so N is second in controlling the eutrophication process by limiting N import into freshwater ecosystems. This is mainly due to the ability of some cyanobacteria to fix atmospheric nitrogen (N2) to fulfil their N requirements [46]. The positive correlation between the TN:TP ratio and Chl-a in our study emphasises that it is not sufficient to focus only on P limitation; both N and P play a crucial role in limiting phytoplankton biomass. Natural N and P concentrations in oligotrophic lakes are low [9], and this is also true for oligotrophic Lake Vransko with the lowest TN:TP ratios, which is in agreement with Elser et al. [47] and Bergström et al. [48]. Lake Vransko is thus N-limited, and the uptake of N can strongly increase the TN:TP ratio, so phytoplankton growth in such lakes can quickly change from a mainly N-limited to a mainly P-limited form [9]. While P often limits phytoplankton biovolume in many lakes, especially deep lakes, N is a better predictor of phytoplankton biomass than P when the N:P ratio is low, as shown by Dolman et al. [49] and Dolman and Wiedner [50]. Jiang and Nakano [51] also suggested that nitrogen plays a greater role than P in freshwater habitats characterised by a low nutrient supply, which is consistent with the nitrogen-limited oligotrophic Lake Vransko. In contrast to the findings of Bergström [9], according to which a low TN:TP ratio is characteristic of lakes with low productivity, Zhou et al. [52] argue that eutrophic lakes are generally characterised by low TN:TP ratios and that higher TN:TP ratios occur more frequently in mesotrophic and oligotrophic lakes. Since the lakes in the above study are highly eutrophic and have high N and P concentrations, their ratio is low, while Lake Vransko, with a low concentration of both nutrients, also has a low ratio. Therefore, with the exception of the above-mentioned Lake Vransko, these results are consistent with the potentially P-limited oligotrophic Lake Kozjak and the predominantly N- and P-limited oligo-mesotrophic lakes Visovac and Oćuša, as well as the mesotrophic lakes Crniševo and Prošće. 

According to nitrogen and phosphorus as the main nutrients for phytoplankton growth, the lakes studied are nutrient-poor lakes and are described as oligotrophic. As the nutrient concentrations are low, the phytoplankton’s need for their uptake is crucial. Jiang and Nakano [51] assumed that phytoplankton has a constant N requirement due to its importance for photosynthesis, while the P requirement can be more flexible due to adaptation and acclimatisation, as the cellular abundance of N in phytoplankton is less plastic than the P content according to Galbraith and Martiny [53]. Phytoplankton subject to dual N and P limitation would therefore have a higher requirement for N than for P, suggesting a greater importance of N for phytoplankton productivity in oligotrophic environments with a low nutrient supply [51]. This is consistent with the importance of TN as one of the determining factors for phytoplankton composition in low-nutrient lakes investigated in our study.

### 3.3. Composition of Phytoplankton FGs and Influence of Environmental Variables

As the results of this study show, the composition of phytoplankton reflects the effects of eutrophication, which is shown graphically in the cluster analysis, in which lakes with similar productivity are grouped together despite different grouping based on environmental parameters. This confirms the role of phytoplankton as one of the most important biological elements in assessing the eutrophication gradient [14,54] and the importance of applying the concept of Reynold’s functional groups in studies of phytoplankton in the environment [18,19,20]. The functional diversity was different for each lake and varied over time, but the coexisting functional groups are characteristic of natural oligotrophic to mesotrophic deep karst lake systems [22], which was also confirmed by the SIMPER analysis of FG composition in this study. The summarised results of this research provide an excellent basis and reference data for future observations of anthropogenic influence and climate change for the purpose of water management of the investigated lakes.

In this study, a comprehensive data set was used to investigate the response of phytoplankton to environmental changes, which will serve as a basis for further observation of changes due to anthropogenic influences and climate change in the lakes studied. The results showed that nutrients and water temperature have a significant influence on the phytoplankton community. These ecological indicators are directly influenced by climate change, which consequently affects the phytoplankton community and its biomass. Dory et al. [55] found that phytoplankton biovolume is more strongly influenced by the effects of temperature than by nutrient availability and also showed that the relative importance of temperature and nutrients for phytoplankton biovolume depends on the trophic status of lakes, with nutrients possibly playing a greater role in oligotrophic lakes, while temperature is more important in mesotrophic lakes. In nutrient-poor lakes, the lack of nutrients may prevent phytoplankton from responding to increasing water temperatures. In contrast, in more nutrient-rich environments, the removal of nutrient limitations increases the sensitivity of phytoplankton to warming. These findings and the results of our study provide a good basis for the further monitoring and investigation of phytoplankton composition and abundance in oligotrophic and mesotrophic lakes.

Although the study was conducted in karst lakes, the results may be generally applicable as the lakes studied are deep and stratified, just like many other lakes around the world with a cosmopolitan phytoplankton community and nitrogen and phosphorus as the main variables [56] to whose concentration changes the phytoplankton responds. The concentrations in the lakes studied are low, so the results obtained are very valuable for research and comparison with meso-oligotrophic lakes, which also have low nutrient levels. In addition, monitoring the composition and abundance of phytoplankton in relation to changing nutrient concentrations is essential for the functioning of freshwater ecosystems. The lakes studied represent largely intact ecosystems that can be used to study changes in the phytoplankton community under the influence of humans and the resulting climate changes.

Bray–Curtis similarity and clustering is a method for analysing beta diversity, and SIMPER analysis reveals characteristic and dominant taxa or functional groups in the categories/lakes studied. Both lack explanatory variables for a deeper understanding of environmental processes. Therefore, an RDA analysis was performed to explain the influence of environmental data on the phytoplankton composition. Regarding the specific nutrients in the two oligotrophic lakes, nitrogen and silicates, their higher concentrations in Lake Kozjak compared to Lake Vransko influenced the differences in phytoplankton community development. The FG composition of Lake Kozjak with codons **B**, **C**, **D** and **P** was more similar to Lake Prošće than to Lake Vransko, as these codons are more tolerant to light deficiency in both lakes than in Lake Vransko. Sensitivity to silicate depletion [18] could also influence the absence of the above-mentioned coda, as the silicate concentration is lowest in Lake Vransko. These factors, together with the higher alkalinity, determine other driving factors for lakes Kozjak and Prošće than for Lake Vransko. Desmids, which belong to codons **T** and **N** and favour environments with low alkalinity and nutrient content [57], are therefore one of the main components of Lake Vransko. 

Cryptophytes in codon **X2** and their mesotrophic character is consistent with the occurrence in lakes Visovac and Oćuša and their tolerance to lower light conditions. The occasional oligo-mesotrophic character of these lakes is also consistent with the chlorophytes and ochrophytes of codon **X3**, which are characteristic of well-mixed oligotrophic environments [19] and occur in lakes Visovac and Oćuša. Both coda have a wide range of tolerance to changes in environmental conditions in these (oligo)mesotrophic lakes, confirming previous results [58,59]. The highest mean SiO_2_ concentrations and the lack of light, especially in summer, most likely contributed to the high biomass and dominance of codon **B** in Lake Visovac, as this codon tolerates less light and is sensitive to Si depletion [18]. Codon **A**, specific for clear, deep lakes with low nutrients [18,19], favoured conditions with more light, especially in the less productive lakes Vransko and Kozjak. 

Dinoflagellates in codon **L0** codominant with cholorophytes in coda **F** and **J**, both specific to mesotrophic lakes with clear epilimnion, favoured the higher water temperature and salinity in the warmest southernmost Mediterranean lakes Oćuša and Crniševo. Nitrogen-fixing cyanobacteria in codon **H1**, which are tolerant to low nitrogen, were found in Lake Crniševo according to their occurrence. Our results are in agreement with the findings of Li et al. [60], where chlorophytes were abundant in slightly brackish lakes (0.8–1.1 salinity). According to Maberly et al. [61], high temperatures are also favourable for the development of chlorophytes (codon **F**) and dinoflagellates (codon **L0**), which is consistent with the occurrence of this coda in our study. Codon **F** was frequently found codominant in lakes with clear epilimnion, but differences between Prošće, Vransko, Oćuša and Crniševo lakes in terms of light availability, temperature and nutrient availability are evident, confirming a wide range of tolerances and sensitivities for the species grouped in codon **F** [18,19,62]. The adaptability of codon **Y** to a wide range of habitats was consistent with the high frequency of occurrence in all lakes studied. Although the driving factor for its growth was the availability of light, its tolerance to low-light conditions with mixotrophic representatives enabled its occurrence during periods of low light [18,19,62,63]. Codon **E** also showed a high occurrence in the studied lakes; most likely, their mixotrophic character played an important role for the high biomass in Lake Oćuša, where the mean Secchi depth is the lowest, and in Lake Prošće, where light availability decreases strongly in summer.

## 4. Materials and Methods

### 4.1. Study Area

**Figure 6:**
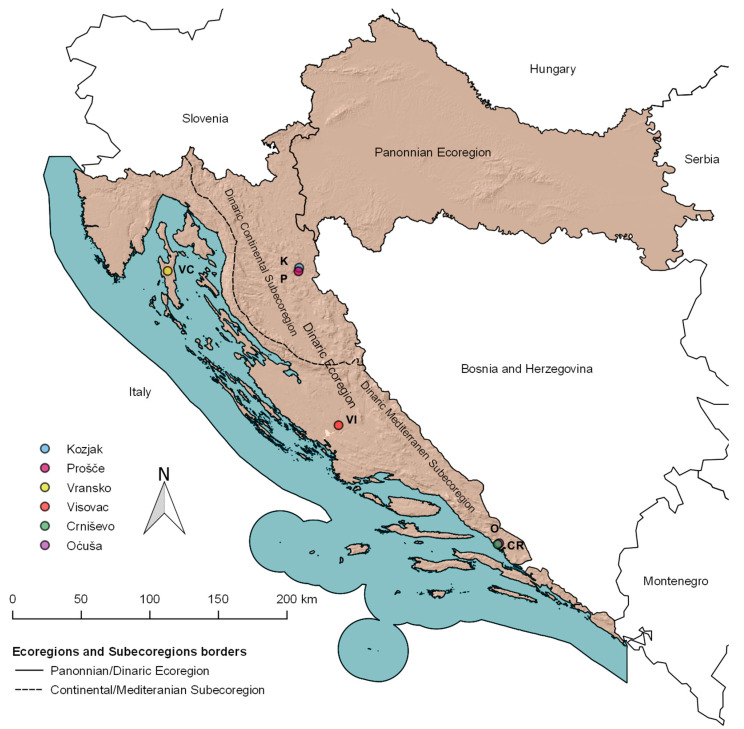
Map of investigated lakes. Lake codes: K—Lake Kozjak; P—Lake Prošće; VC—Lake Vransko; VI—Lake Visovac; CR—Lake Crniševo; and O—Lake Oćuša.

The Dinaric and Pannonian ecoregions represent two distinct ecological and geographical areas in Croatia (Figure 6). All six natural deep karst lakes, each covering an area of more than 0.5 km^2^, are located in the Dinaric ecoregion. The Plitvice Lakes, located in the Dinaric Continental Subecoregion, were formed by a combination of tectonic shifts, the development of tufa formations and the presence of travertine barriers. A total of 16 barrage lakes have formed in the region, with Lake Kozjak being the deepest and largest, closely followed by Lake Prošće [4]. Both lakes have dimictic features typical of mountain lakes influenced by a continental climate. The other lakes are located in the Dinaric Mediterranean Subecoregion. The formation of Lake Visovac is a remarkable example of the lenticular dilation of the Krka River, which is also a lake formed by tufa formation [22]. The last three lakes are cryptodepressions on the Adriatic coast. The deepest, Lake Vransko on the island of Cres, was formed during the transition from the Pliocene to the Pleistocene [64]. Lakes Crniševo and Oćuša are part of the connected Baćina Lakes complex. Lake Crniševo has slightly brackish characteristics due to underground brackish water springs and saltwater intrusion due to its proximity to the sea. Lake Oćuša, the largest lake within the complex, although connected to Lake Crniševo, is a freshwater lake due to freshwater springs and as there is no water exchange between them [65]. The geographical coordinates and physical characteristics of these lakes are described in detail in a previously published article [23].

### 4.2. Sampling and Sample Analysis

The water samples were taken monthly during the growing season (April to September) in five to seven years between 2013 and 2022, with different dynamics depending on the water quality monitoring plan. A total of 209 phytoplankton samples were collected at the deepest point of each lake [66]. Both the phytoplankton samples and the samples for analysing the environmental parameters were collected as composite samples using a Uwitec water sampler. During thermal stratification, the composite samples were taken from the euphotic zone or the epilimnion, whichever was deepest, during the non-stratification period to a maximum depth of 20 metres. Immediately after collection, the phytoplankton samples were stored in 250 mL glass bottles and preserved with acidic Lugol’s solution for further microscopic analysis. The phytoplankton was counted and identified according to the Utermöhl [67] method using an inverted microscope (Zeiss Axio Observer Z1 or Olympus IX 51 with DIC) at 400×, 200× and 100× magnification. The sedimentation units (unicellular, coenobium, filament or colony) were counted in random counting fields or transects until 400 sedimentation units were counted at 400× magnification, ensuring a counting error of less than 10% [68]. The individual cells were measured and their biovolume approximated to the nearest regular geometric shape. Biovolumes were then calculated by determining the median size of up to 30 randomly selected cells within each taxon and multiplying this value by the observed taxon abundance. Biomass (fresh weight) was obtained from the biovolumes and used for subsequent analyses, with a conversion rate of 1 mm^3^ L^−1^ equalling 1 mg L^−1^ [69,70]. Additional identification of diatoms was performed using permanent slides prepared by cleaning the samples with warm hydrochloric acid and hydrogen peroxide and then mounting them using Naphrax solution [71]. Diatoms were identified at 1000× magnification using an upright microscope (Zeiss Axio Observer Z1 or Olympus BX51 with DIC). After analysis, the names were revised in accordance with Algaebase [72], and the taxa were categorised into functional groups [18,19,20].

The measurement of environmental parameters is described in Stanković et al. [13].

### 4.3. Data Analysis

The map of the study area was created with QGIS 3.34 [73]. The cluster analysis of the environmental variables in the lakes based on Euclidean distance was carried out using Primer 7 software [74]. The chlorophyll a concentration (Chl-a), Secchi depth, total phosphorus, total nitrogen concentration and molar TN:TP ratio were displayed as boxplots in SCImago Graphica [75]. The Secchi depth, Chl-a, TP and TN were categorised into trophic status according to Miliša et al. [76], who modified OECD [77] boundaries to local conditions.

The Spearman correlation coefficient was used in this study to examine the relationships between phytoplankton biomass (including Chl-a and total biomass) and environmental variables in the lakes, using IBM SPSS Statistics [78]. Primer 7 software was also used for the cluster analysis of FG composition based on Bray–Curtis similarity. Prior to analysis, biomass was square-root-transformed.

Canonical redundancy analysis (RDA) was performed to evaluate the relationship between phytoplankton FG composition and environmental parameters in each lake. The analysis was performed using CANOCO 5.15 software [79]. All FGs, 209 samples and all environmental variables were included in the analysis. The ordination results were presented using correlation triplots. Phytoplankton biomass data were log-transformed, while environmental data were normalised prior to analysis. A draftman’s plot was used to identify and remove variables with significant autocorrelation. Forward selection was then applied to data sets with response variables and environmental descriptors as explanatory variables. Only variables that showed significance at the level of *p* ≤ 0.05 (999 permutations) were selected for further analysis.

## Figures and Tables

**Table 1:** Minimum, maximum and mean values of physical and chemical parameters for investigated lakes in the period from 2013 to 2022. Abbreviations used in text are in square brackets.

Parameter	Kozjak		Prošće		Vransko	
	Min-Max	Mean	Min-Max	Mean	Min-Max	Mean
Secchi visibility (m)	5.3–14.7	8.5	3.6–10.7	5.4	7.8–15.5	11.0
Temperature (°C)	7.6–18.8	13.3	8.4–18.1	12.8	9.9–21.7	17.7
pH	7.9–8.4	8.3	7.7–8.4	8.2	7.9–8.4	8.3
Alkalinity (mg CaCO_3_ L^−1^)	202.0–236.0	214.0	214.0–249.0	230.0	102.0–139.0	112.0
Dissolved oxygen (mg L^−1^)	10.0–13.7	11.7	8.5–15.0	11.2	8.5–12.7	10.7
Oxygen saturation (%)	96.8–146.9	113.2	79.8–159.1	106.7	91.1–134.3	115.5
Conductivity (µS cm^−1^) 25 °C	359.0–419.0	393.5	387.0–443.0	428.5	397.0–475.0	436.0
Salinity (‰)	0.10–0.14	0.13	0.00–0.15	0.13	0.11–0.15	0.13
Total nitrogen [TN] (mg N L^−1^)	0.420–0.990	0.685	0.520–0.940	0.740	0.100–0.310	0.100
Total phosphorus [TP] (mg P L^−1^)	0.002–0.059	0.013	0.002–0.038	0.018	0.002–0.044	0.014
Molar TN:TP ratio	10.5–493.7	62.5	19.4–523.2	45.4	2.5–228.4	14.8
Biological oxygen demand [BOD] (mg O_2_ L^−1^)	0.3–1.8	0.8	0.3–1.7	1.0	0.3–1.5	0.6
Chemical oxygen demand [COD] (mg O_2_ L^−1^)	0.4–2.2	1.2	0.4–3	1.4	0.4–2.4	1.4
Total organic carbon [TOC] (mg C L^−1^)	0.69–2.22	1.02	0.75–2.09	1.08	1.19–2.93	1.67
Silicates [SiO_2_] (mg L^−1^)	0.57–3.41	1.60	0.35–4.96	1.58	0.13–1.39	0.35
Chlorophyll *a* [Chl-a] (µg L^−1^)	0.35–2.72	1.29	0.5–8.14	4.01	0.35–2.06	0.50
Total biomass (mg L^−1^)	0.12–2.05	0.46	0.35–4.65	1.49	0.21–1.21	0.38
**Parameter**	**Visovac**		**Crniševo**		**Oćuša**	
	**Min-Max**	**Mean**	**Min-Max**	**Mean**	**Min-Max**	**Mean**
Secchi visibility (m)	3.2–8.2	5.3	1.0–6.5	4.8	3.0–8.0	4.4
Temperature (°C)	13.2–23.0	18.0	11.8–27.3	21.4	14.2–27.0	22.6
pH	6.7–8.4	8.1	7.6–8.4	8.1	7.5–8.7	8.1
Alkalinity (mg CaCO_3_ L^−1^)	171.0–245.0	210.1	139.0–368.8	171.0	112.0–201.0	150.0
Dissolved oxygen (mg L^−1^)	6.9–13.6	10.3	8.3–13.0	10.6	5.9–12.7	9.9
Oxygen saturation (%)	73.4–162.3	107.3	88.1–140.1	117.5	66.9–153.6	111.3
Conductivity (µS cm^−1^) 25 °C	482.0–895.0	535.0	718.0–2930.0	1853.0	372.0–600.0	457.0
Salinity (‰)	0.16–0.31	0.20	0.29–1.50	0.88	0.11–0.30	0.22
Total nitrogen [TN] (mg N L^−1^)	0.220–0.753	0.350	0.240–1.010	0.385	0.100–0.909	0.429
Total phosphorus [TP] (mg P L^−1^)	0.003–0.033	0.012	0.002–0.045	0.009	0.002–0.036	0.007
Molar TN:TP ratio	11.7–150.3	33.9	11.5–694.1	48.6	11.4–316.1	39.6
Biological oxygen demand [BOD] (mg O_2_ L^−1^)	0.3–2.4	0.8	0.1–2.4	1.2	0.1–2.7	0.9
Chemical oxygen demand [COD] (mg O_2_ L^−1^)	0.3–3.5	1.1	0.3–5.4	2.6	0.3–2.8	1.4
Total organic carbon [TOC] (mg C L^−1^)	0.65–1.59	1.07	1.50–3.70	2.42	0.82–2.9	1.28
Silicates [SiO_2_] (mg L^−1^)	0.40–4.52	1.95	0.17–2.11	0.70	0.28–3.54	1.52
Chlorophyll *a* [Chl-a] (µg L^−1^)	1.18–7.39	2.90	1.5–11.32	2.42	0.51–5.67	3.22
Total biomass (mg L^−1^)	0.38–4.70	1.27	0.22–6.87	0.82	0.22–3.81	0.86

**Table 2:** Spearman’s Rho correlations (two-tailed) for relationships between phytoplankton biomass (including chlorophyll *a* concentration and total biomass) and environmental variables in all lakes. Correlation is significant at * *p* ≤ 0.05 level and ** *p* ≤ 0.01 level in bold; n.s. not significant; total number of samples = 209.

Environmental Variables	Lakes (*n* = 209)	
	Chlorophyll *a*	Total Biomass
Light availability	−0.741 **	−0.571 **
Alkalinity	0.307 **	0.368 **
Conductivity	0.207 **	0.178 **
pH	−0.276 **	−0.212 **
Salinity	0.204 **	0.150 *
Temperature	n.s.	n.s.
Biological oxygen demand	0.331 **	0.256 **
Chemical oxygen demand	0.165 *	n.s.
Dissolved oxygen	−0.152 *	n.s.
Oxygen saturation	−0.159 *	n.s.
Total nitrogen	0.222 **	0.305 **
Total phosphorus	n.s.	0.180 **
Total organic carbon	n.s.	−0.150 *
Silicon dioxide	0.252 **	0.224 **
TN:TP (mol)	0.147 *	n.s.

**Table 3:** Descriptive phytoplankton functional groups coda determined by SIMPER analysis are presented as a contribution to the similarity of all samples for each lake (SIMPER Ctb./%) over the entire study period from 2013 to 2022.

	Kozjak (*n* = 42)	Prošće (*n* = 42)	Vransko (*n* = 36)	Visovac (*n* = 30)	Crniševo (*n* = 30)	Oćuša (*n* = 29)
FG’s	%	%	%	%	%	%
A	28.58	6.49	24.31	-	17.04	31.88
B	14.77	24.3	3.87	44.04	-	-
D	6.42	3.66	-	-	-	-
T	-	-	8.22	-	-	-
X3	-	-	-	8.85	-	-
X2	12.95	12.18	7.52	15.27	12.22	23.09
X1	-	-	-	-	6.59	-
E	9.37	16.38	10.25	-	-	10.73
Y	8.21	14.47	-	-	-	3
F	3.63	9.53	5.13	9.01	15.46	8.54
J	-	-	-	-	-	5.49
L0	7.19	4.46	32.6	13.13	39.16	9.92

**Table 4:** Results of the redundancy analysis (RDA) between FGs and environmental parameters for deep karst lakes. ^a^ Axis summary statistics of the two extracted canonical axes and the percentage of variance explained by the RDA ordination; ^b^ correlation of the environmental variables with the ordination axes; explanatory variables at *p* ≤ 0.05 significance level (999 permutations) in the forward selection are in bold with *p*-value. Codes of variables: T—water temperature; Sal—salinity; Secchi—Secchi depth; Alk—alkalinity; TN—total nitrogen; SiO_2_—silicates; and TOC—total organic carbon.

	Axis 1	Axis 2
*Axis summary statistics and variance in species data* ^a^		
Eigenvalues	0.163	0.058
FG–environment correlations	0.844	0.714
Cumulative percentage variance		
Of FG data	16.3	22.1
Of FG–environment relation	56.1	76.1
*Correlations of environmental variables and redundancy axes* ^b^		
Temp	0.611	0.202
Sal	0.512	0.24
Secchi	−0.034	−0.597
Alk	−0.568	0.363
TN	−0.653	0.115
SiO_2_	−0.408	0.323
TOC	0.502	−0.102

## Data Availability

The raw data supporting the conclusions of this article are available upon request because they are results from monitoring.

## Supplementary Materials

The following supporting information can be downloaded at https://www.mdpi.com/article/10.3390/plants13162252/s1, Table S1: Complete list of phytoplankton taxa in the investigated lakes.
